# Detection of Extensive Cross-Neutralization between Pandemic and Seasonal A/H1N1 Influenza Viruses Using a Pseudotype Neutralization Assay

**DOI:** 10.1371/journal.pone.0011036

**Published:** 2010-06-09

**Authors:** Béatrice Labrosse, Mathieu Tourdjman, Raphaël Porcher, Jérôme LeGoff, Xavier de Lamballerie, François Simon, Jean-Michel Molina, François Clavel

**Affiliations:** 1 Institut Universitaire d'Hématologie, Université Paris Diderot, Paris, France; 2 Inserm U941, Paris, France; 3 Service des Maladies Infectieuses, Hôpital Saint Louis, Assistance Publique-Hôpitaux de Paris, Paris, France; 4 Département de Biostatistique, Hôpital Saint Louis, Assistance Publique-Hôpitaux de Paris, Paris, France; 5 Laboratoire de Microbiologie, Hôpital Saint Louis, Assistance Publique-Hôpitaux de Paris, Paris, France; 6 Université Aix-Marseille 2 and Institut de Recherche pour le Développement, Marseille, France; Tsinghua University, China

## Abstract

**Background:**

Cross-immunity between seasonal and pandemic A/H1N1 influenza viruses remains uncertain. In particular, the extent that previous infection or vaccination by seasonal A/H1N1 viruses can elicit protective immunity against pandemic A/H1N1 is unclear.

**Methodology/Principal Findings:**

Neutralizing titers against seasonal A/H1N1 (A/Brisbane/59/2007) and against pandemic A/H1N1 (A/California/04/2009) were measured using an HIV-1-based pseudovirus neutralization assay. Using this highly sensitive assay, we found that a large fraction of subjects who had never been exposed to pandemic A/H1N1 express high levels of pandemic A/H1N1 neutralizing titers. A significant correlation was seen between neutralization of pandemic A/H1N1 and neutralization of a standard seasonal A/H1N1 strain. Significantly higher pandemic A/H1N1 neutralizing titers were measured in subjects who had received vaccination against seasonal influenza in 2008–2009. Higher pandemic neutralizing titers were also measured in subjects over 60 years of age.

**Conclusions/Significance:**

Our findings reveal that the extent of protective cross-immunity between seasonal and pandemic A/H1N1 influenza viruses may be more important than previously estimated. This cross-immunity could provide a possible explanation of the relatively mild profile of the recent influenza pandemic.

## Introduction

The influenza A/H1N1 virus of swine origin, first isolated in April 2009 in Mexico and the United States, has now been spreading worldwide, causing the first influenza pandemic in more than 40 years [1], [2], [3]. Although this virus generally causes only mild clinical symptoms, a number of severe cases have been described, prompting rapid development of specific vaccine preparations for mass vaccination [4], [5], [6], [7]. In this context, however, the extent that preexisting immunity to seasonal influenza viruses can provide cross-protection against pandemic A/H1N1 remains uncertain. Using a highly sensitive pseudovirus neutralization (PN) assay, we have examined the ability of sera from 107 subjects with no history of exposure to pandemic A/H1N1 to cross-neutralize this new influenza strain. We correlated these cross-neutralization titers with neutralization of a standard seasonal A/H1N1 strain, and evaluated the impact of the subject's age and history of recent seasonal influenza vaccination on cross-neutralization.

## Results

None of the 107 subjects had reported recent symptoms evocative of influenza infection in the weeks preceding serum sampling, a period during which the pandemic A/H1N1 influenza had barely reached epidemic levels (181 cases/100,000 persons) in France [8]. The median age of the subjects was 44 years (range 20–90 years), 75/107 were female, and 54/107 reported vaccination by trivalent seasonal influenza vaccine during the 2008/2009 epidemic season. In order to obtain a batch of positive control sera expressing high titers of neutralizing antibodies against the pandemic strain, a group of 10 subjects were vaccinated against pandemic A/H1N1 by a single injection of an adjuvanted, split virus vaccine preparation containing 3.75 µg of viral hemagglutinin (Pandemrix®, Glaxo SmithKline). Serum from these 10 subjects was collected before and 3–4 weeks after this inoculation and tested for cross-neutralization of pandemic and seasonal A/H1N1, alongside the 107 other serum samples.

Reproducibility of the PN assay was evaluated by test-retest analysis on all tested samples, yielding satisfactory intraclass correlation coefficients (ICC) of 0.840 (95%CI 0.774 to 0.888) and 0.846 (95%CI 0.782 to 0.892) for the pandemic and seasonal viruses, respectively. The specificity of the assay was validated by comparing neutralization titers of pre- and post-pandemic A/H1N1 vaccination serum samples from the 10 subjects described above. The results of this analysis are shown on table 1. Vaccination against the pandemic strain increased pandemic A/H1N1 neutralizing titers in the PN assay by a geometric mean of 12.8-fold (95%CI 4.9 to 33.5, p<0.0001 *vs* baseline), while seasonal vaccinal Brisbane A/H1N1 neutralizing titers only increased by 1.2-fold (95%CI 0.31 to 4.5). As a control, the same pairs of samples were also tested using a standard hemagglutination inhibition (HI) assay measuring antibodies to the pandemic A/H1N1 virus, which showed clear but relatively modest increases in antibody titers, when compared to the increases measured by the PN assay. Using the HI assay, baseline titers were low (titer <40 in 9/10 samples) and increased following vaccination in all subjects, but only 7/10 post-vaccination sera had a titer ≥80 (Table 1). A significant correlation, however, was seen between the PN and HI titers in sera from subjects having received the pandemic A/H1N1 vaccine (Spearman r_s_ = 0.96, 95%CI 0.85 to 0.99, p<0.0001; data not shown).

**Table 1:** Changes in neutralizing and HI titers after pandemic A/H1N1 vaccination.

	Neutralizing titer, seasonal		Neutralizing titer, pandemic		HI titer, pandemic	
Subject	Baseline	Fold-change after pandemic vaccine	Baseline	Fold-change after pandemic vaccine	Baseline	After pandemic vaccine
#1	10	35.7	38	31.4	<40	40
#2	43	0.41	166	14.8	<40	160
#3	200	1.62	461	9.8	<40	160
#4	316	2.03	425	21.7	80	320
#5	387	0.79	111	22.0	<40	160
#6	502	3.09	409	5.4	<40	80
#7	596	0.65	265	90.0	<40	320
#8	625	0.30	409	2.9	<40	40
#9	1505	0.29	223	3.2	<40	40
#10	1640	0.63	965	12.3	<40	320
Geometric mean (95%CI)		1.2 (0.31 to 4.5)		12.8 (4.9 to 33.5)*		

*p<0.0001 vs baseline.

**Figure 1:**
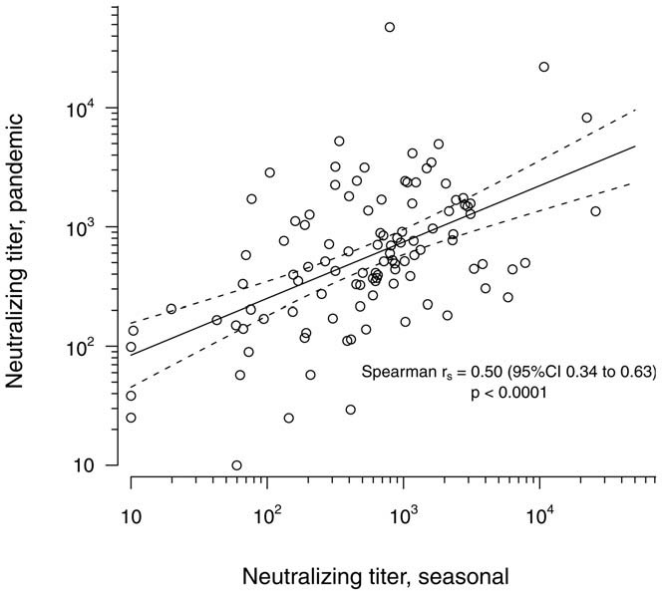
Correlation between pandemic A/H1N1 and seasonal A/H1N1 neutralizing titers. Neutralizing titers are expressed as the log_10_ of the geometric mean of titers calculated as described in Materials and Methods in two independent experiments. Linear regression curve (continuous line) and 95%CI (dotted line) are presented. The correlation coefficient and P value were calculated by Spearman test.

**Figure 2:**
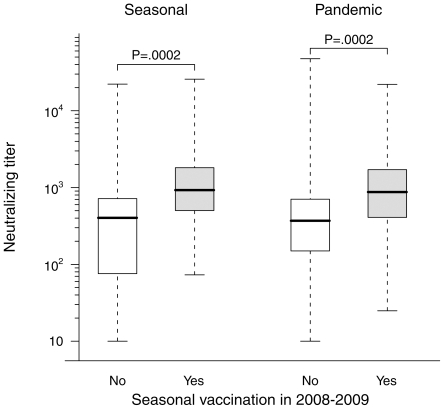
Comparison of pandemic A/H1N1 neutralizing titers in subjects vaccinated or not against seasonal influenza during the 2008–2009 epidemic season. Box and whiskers plots present the median, first and third quartile of the distribution (box) and outer whiskers extend the whole range of data. P values are from a Wilcoxon rank sum test.

**Figure 3:**
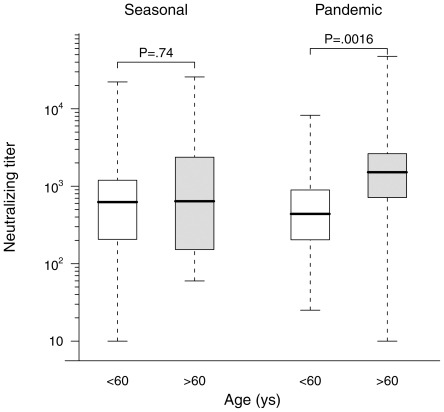
Comparison of pandemic A/H1N1 neutralizing titers in subjects according to age. Symbols and P values are the same as those on figure 2.

The range of pandemic A/H1N1 neutralizing antibody titers by PN assay in subjects who had no known exposure to this virus was remarkably wide, and often reached surprisingly high levels. Only 9 sera titrated <100 while 43 titrated between 100 and 500, 45 titrated between 500 and 2500, and 10 sera titrated >2500, including two sera with titers >20,000. A comparable range of neutralizing titers was observed against the seasonal Brisbane A/H1N1 strain. Moreover, as illustrated on figure 1, a significant correlation was observed between the neutralizing titers against the two strains (Spearman r_s_ = 0.50, 95%CI 0.34 to 0.63, p<0.0001). This finding strongly suggests that previous exposure to seasonal influenza viruses and/or previous seasonal influenza vaccination had conferred some level of protective immunity against the new pandemic A/H1N1 strain in many of the study subjects. We therefore compared the pandemic A/H1N1 and seasonal Brisbane vaccinal A/H1N1 neutralizing titers in sera from subjects having reported inoculation by a seasonal trivalent influenza vaccine during the 2008–2009 epidemic season to titers measured in sera from subjects who did not report such vaccination. As shown on figure 2, this comparison revealed significantly higher neutralizing titers to both the seasonal and pandemic strains in vaccinated subjects (p = 0.002 for both comparisons), indicating that in subjects who had never been exposed to pandemic A/H1N1 antigens, vaccination against seasonal influenza during the previous year had elicited antibodies with the ability to cross-neutralize the pandemic strain. We also examined the effect of age on the presence of cross-neutralizing antibodies to pandemic A/H1N1 in the same population of subjects. As shown on figure 3, sera from patients older than 60 had significantly higher neutralizing titers to the pandemic virus than younger patients, but no difference was seen when the two age classes were compared for neutralizing titers against the seasonal virus.

## Discussion

Based on a highly sensitive pseudovirus neutralization assay, we found that a large proportion of sera from a panel of subjects with no previous exposure to the recently emerged pandemic A/H1N1 influenza strain expressed high titers of neutralizing antibodies against this new virus. Furthermore, our data show that these neutralizing titers correlate significantly with neutralizing titers against a seasonal A/H1N1 virus measured in parallel, and that the pandemic A/H1N1 neutralizing titer is significantly higher in subjects who had been incoculated by a seasonal trivalent influenza vaccine during the previous year. These data are in apparent contradiction with the conclusions of a retrospective study reported by Hancock et al. [9], where only minor changes in pandemic A/H1N1 neutralizing titers were measured following seasonal influenza vaccination. We believe that the difference between our observations and those of Hancock et al. owes largely to the sensitivity and reproducibility of the PN assay used in our study, which allows measurement of neutralizing titers over a wide range of values and with high discrimination between titers of different levels.

Our findings are in line with several reports suggesting that some level of A/H1N1 cross-neutralization could be induced by seasonal influenza viruses. First, in some of the studies evaluating the safety and immunogenicity of vaccine preparations against pandemic A/H1N1, high titers of HI antibodies were seen in subjects before vaccination [10], [11], [12], and at least one of these studies provided indications of higher HI or neutralizing titers in subjects with a history of recent vaccination against seasonal influenza A [12]. Second, a recent report revealed that priming with a seasonal A/H1N1 inoculation before pandemic A/H1N1 vaccination could enhance protection of ferrets against pandemic H1N1 [13]. Third, strong evidence exists that several epitopes in HA could promote cross-immunity between distant strains of influenza A viruses [14], [15] and that subjects with previous immunity to seasonal A/H1N1 may delelop milder form of pandemic A/H1N1 infection [16]. Taken together, these and our results strongly suggest that some extent of cross-protection against pandemic A/H1N1 may exist in a proportion of subjects in the general population, and that prior vaccination against seasonal A/H1N1 explains, at least in part, this cross-immunity.

Several factors could explain the high levels of cross-immunity observed in our study. The particularly strong cross-immunity seen in subjects older than 60 (Fig 3) is noteworthy. It is in agreement with a recent study evaluating HI and microneutralization titers in sera obtained in 2008 [17] and with with a study examining HI titers in 2009 sera [18]. Interestingly, we observe that the higher pandemic A/H1N1 neutralizing titers measured in these subjects are not accompanied by higher seasonal A/H1N1 titers, suggesting that they may have had past exposure to A/H1N1 variants that share more antigenic similarity to the new pandemic A/H1N1 virus than to recently circulating seasonal A/H1N1 strains. Nonetheless, since high pandemic neutralizing tirers were also measured in younger subjects and particularly in subjects having received seasonal A/H1N1 vaccination during the 2008–2009 season, our data indicate that the observed cross-immunity can also be partly explained by immunity against seasonal A/H1N1 strains, in spite of the wide genetic distance between recent A/H1N1 influenza strains and the new pandemic strain. Of note, while vaccination against seasonal A/H1N1 was significantly associated with higher pandemic neutralizing titers, there was no increase in seasonal neutralizing titers in the 10 control subjects who were vaccinated against pandemic A/H1N1. The mechanisms underlying this “asymmetric” cross-immunity have yet to be understood.

The extent that the cross-immunity observed in this study has had any impact on the relatively mild profile of the current pandemic remains to be determined, and at this stage, our results do not invalidate the clinical and epidemiological utility of mass vaccination against pandemic A/H1N1 influenza virus. In fact, the cross-immunity described here could explain, at least in part, the strong immunological response to a single dose of pandemic A/H1N1 vaccine in adults.

## Materials and Methods

Serum samples from 107 subjects were obtained between Aug 18 and Oct 10, 2009 from primary care personnel and patients at Hôpital Saint Louis, Paris, France. Written informed consent was obtained for all patients, and the study was approved by the Paris Saint-Louis ethical committee.

The pseudovirus neutralization assay used in this study is based on infectivity of pseudotyped HIV-1 particles expressing influenza HA proteins. Briefly, subconfluent monolayers of human 293-T cells in 6-well plates were co-transfected with 0.7 µg of the HIV-1 pNL4-3-ΔENV-LucR vector and 0.5 µg of the pVITRO2-blasti-mcs-*tat* plasmid, expressing the influenza HA protein either from a vaccinal strain of seasonal A/H1N1 (A/Brisbane/59/2007) or from pandemic A/H1N1 (A/California/04/2009), and grown in the presence of 5 mU/mL of soluble *Clostridium perfringens* neuraminidase. After 48 hours, the pseudovirus-containing supernatants were harvested and volumes of supernatant expressing an infectious titer of 10^4^ RLU (Relative Light Unit) were incubated with twofold dilutions of decomplemented test serum (starting dilution 1∶20) for 1 hour at 37°C. Subsequently, 200 µL of virus-antibody mixture were transferred to MDCK-SIAT1 target cell cultures in quadruplicate in 96-well plates. The inhibitory effect of serum on pseudovirus infectivity was measured 48 h later by fitting the percent inhibition of RLU values to a sigmoid dose-response curve with variable slope, using the GraphPad *Prism* 4.0b software. The neutralizing titer was defined as the reciprocal of the serum dilution required to reduce viral infectivity by 50% and expressed as the geometric mean from two independent experiments.

Titration of antibodies to pandemic A/H1N1 by hemagglutination inhibition (HI) was performed by a standard assay using human group O erythrocytes and non-inactivated pandemic A/H1N1 virus.

Correlations and comparisons between neutralizing titers were done based on the log_10_ values of the titers. Correlations between neutralizing titers were examined by calculating the Spearman correlation coefficient; comparisons of neutralizing titers before and after pandemic A/H1N1 vaccination were done using the Wilcoxon signed rank-sum test; comparison of neutralizing titers between subject groups were performed using the Wilcoxon rank-sum test.
